# Comparison of the sealing ability for various bioceramic materials as root-end fillings

**DOI:** 10.6026/973206300223096

**Published:** 2026-05-31

**Authors:** Neel Doshi, Divyashree Prasad, Suyash Pratap Singh, Ilham Aliya, Aswiyaa Muhamed Ilyas, Sarita V Singh, Rubeena Naaz, Kafeel Ahmed Md

**Affiliations:** 1 Department of Conservative Dentistry and Endodontics, Bharati Vidyapeeth Dental College and Hospital, Pune, Maharashtra, India; 2 Department of Periodontics, MES Dental College and Hospital, Malappuram, Kerala, India; 3 Department of Conservative Dentistry and Endodontics, Institute of Dental Sciences, Bareilly, Uttar Pradesh, India; 4 Department of Dentistry, Ashirwad Dental Clinic, Bengaluru, Karnataka, India; 5 Department of Dentistry, Dynasty Clinic, Dubai, United Arab Emirates; 6 Department of Dentistry, SRK Dental Clinic, Hyderabad, Telangana, India; 7 Department of Periodontology and Implantology, MNR Dental College and Hospital, Sangareddy, Telangana, India

**Keywords:** Root-end filling, bioceramics, microleakage, mineral trioxide aggregate (MTA), biodentine, and apical seal

## Abstract

The ideal root-end filling material should provide an excellent apical seal, yet the sealing performance of newer bioceramic materials remains uncertain. Therefore, it is of interest to evaluate the sealing ability of four root-end filling materials on 70 extracted single-rooted human teeth prepared under standardized conditions. The specimens were randomly assigned to mineral trioxide aggregate, Biodentine, Bioceramic Putty and Bio-C Repair groups and sealing was assessed by dye penetration under a stereomicroscope. Statistical analysis showed that the newer bioceramic materials sealed better than conventional MTA, with Bioceramic Putty showing the best performance. Thus, we support the clinical potential of bioceramic putty as a promising root-end filling material.

## Background:

The success of endodontic therapy is dependent on the root canal system being free of microorganisms and hermetic seal to inhibit re-infection. But in the failure of conventional root canal therapy, periapical surgery requiring root-end resection and retrograde filling is required to obtain apical sealing and periapical healing [1]. Sealing ability is one of the most important properties of an ideal root-end filling material, alongside biocompatibility, dimensional stability, antimicrobial activity, and the ability to set in the presence of moisture because inadequate sealing permits microleakage that can lead to chronic periapical pathology and treatment failure, a point emphasized by recent comparisons of sealing performance among root-end filling materials [2]. Root-end fillings traditionally were done with different materials like amalgam, intermediate restorative material (IRM) and glass ionomer cement. Nevertheless, these materials proved to have weak sealing and biocompatibility properties. The advent of Mineral Trioxide Aggregate (MTA) was a breakthrough in the field of endodontics because it has better sealing capabilities and biological features [3]. MTA is a calcium silicate-based product that was deemed the gold standard in the area of root-end fillings.

Its capacity to offer an effective apical seal and enhance tissue regeneration has been demonstrated in several studies, but its disadvantages that include long setting time, poor handling properties and expensive nature have resulted in alternative bioceramic materials [4, 5]. More recently, there are newer bioceramic materials like Biodentine, EndoSequence Bioceramic Root Repair Material (BC-RRM) and Bio-C Repair. These materials have better handling characteristics, shorter setting durations and better bioactivity. They are hydrophilic and may also form hydroxyapatite when they are set thus enhancing the bond between dentin and the filling material [6, 7]. In-vitro studies have been performed comparing the sealing capacity of these materials using a variety of methodologies, including dye penetration, fluid filtration and bacterial leakage models. As an example, it was observed that Biodentine can have a high or equal sealing capacity to MTA, but this varies with experimental circumstances [8]. On the same note, more recent bioceramic alloys like BC-RRM and Bio-C Repair have shown encouraging outcomes in minimizing microleakage and sealing properties in comparison to traditional alloys [9]. The leakage studies conducted using bacteria also underscore the significance of selecting the material since significant variations in the seal efficacy have been witnessed between MTA and other bioceramic material when put under microbial challenge conditions [10]. Therefore, it is of interest to compare and contrast sealing characteristics of different bioceramics as root-end fillings using a common methodology.

## Materials and Methodology:

## Study design:

This study was designed as an *in vitro* experimental study to evaluate and compare the sealing ability of different bioceramic root-end filling materials using a dye penetration method.

## Sample selection:

A total of 70 freshly extracted human single-rooted teeth with fully formed apices were selected for the study.

## Inclusion criteria:

[1] Single straight root canals

[2] Fully developed apices

[3] Intact roots without cracks or fractures

[4] No previous endodontic treatment

## Exclusion criteria:

[1] Teeth with resorption

[2] Calcified canals

[3] Open apices

[4] Root caries or structural defects

After extraction, all teeth were cleaned of soft tissue debris and calculus and stored in 0.1% thymol solution until use.

## Sample preparation:

[1] The crowns were decoronate at the cemento-enamel junction using a diamond disc to standardize root length (∼15 mm).

[2] Working length was established 1 mm short of the apex.

[3] Root canals were instrumented using rotary NiTi files up to size F3 (Protaper system) with irrigation using:

1) 3% sodium hypochlorite

2) 17% EDTA

[4] Final irrigation was performed with distilled water to remove any chemical remnants.

The canals were then dried and obturated using gutta-percha with resin-based sealer by lateral condensation technique.

## Apical resection and retrograde preparation:

[1] Apical resection of 3 mm of the root end was performed at 90° to the long axis of the tooth using a diamond disc.

[2] A 3 mm deep retrograde cavity was prepared using ultrasonic retro tips.

This standardized preparation is essential as it improves sealing and reduces apical leakage. 

## Grouping of samples:

The 70 samples were randomly divided into four groups:

[1] Group I (n = 18): Mineral Trioxide Aggregate (MTA)

[2] Group II (n = 18): Biodentine

[3] Group III (n = 17): Bioceramic Putty (EndoSequence BC-RRM)

[4] Group IV (n = 17): Bio-C Repair / equivalent

## Placement of root-end filling materials:

[1] Each material was mixed according to the manufacturer's instructions.

[2] The retrograde cavities were filled using micropluggers.

[3] Care was taken to avoid voids and ensure proper condensation.

[4] All samples were stored in 100% humidity at 37°C for 24-48 hours to allow complete setting of materials.

## Surface coating:

[1] The entire root surface was coated with two layers of nail varnish, except for the apical 1 mm area.

[2] This step prevents dye penetration from surfaces other than the apex.

## Dye penetration test:

[1] Samples were immersed in 2% methylene blue dye solution (or rhodamine B) for 24-48 hours.

[2] Dye penetration is a widely accepted method to evaluate microleakage and sealing ability in endodontic studies.

## Sectioning and evaluation:

[1] After dye exposure, the teeth were rinsed and dried.

[2] Each specimen was sectioned longitudinally in a buccolingual direction using a diamond disc.

[3] The extent of dye penetration was evaluated under a stereomicroscope / confocal microscope at appropriate magnification.

The linear extent of dye penetration was measured in millimetres from the apex toward the canal.

## Outcome measure:

[1] The primary outcome was apical microleakage, measured as:

[2] Linear dye penetration (mm)

[3] Mean leakage values for each group

[4] Lower dye penetration indicated better sealing ability.

## Statistical analysis:

Data were tabulated and analyzed using SPSS software (version 26.0, IBM Corp., Armonk, NY, USA). One-way ANOVA was used to compare mean microleakage between groups. Post hoc Tukey test was applied for pairwise comparison. Significance level was set at p < 0.05

## Results:

A total of 70 samples were evaluated for apical microleakage using dye penetration. The highest percentage of no leakage (Score 0) was observed in BC-RRM (47.1%), followed by Bio-C Repair (41.2%). MTA showed the highest percentage of severe leakage (22.2%), indicating inferior sealing ability. Overall, newer bioceramic materials demonstrated reduced microleakage compared to MTA (Table 1). The lowest mean microleakage was observed in BC-RRM (0.92 mm). MTA exhibited the highest leakage (1.82 mm). The difference between groups was statistically highly significant (p < 0.001), indicating a real difference in sealing ability (Table 2). Significant differences were seen between MTA and all other materials. BC-RRM versus Bio-C Repair showed no statistically significant difference (p >0.05). This suggests that both newer premixed bioceramics perform similarly and better than conventional materials (Table 3).

## Discussion:

The current paper has assessed the sealing capacity of four bioceramic root-end fillings materials through the dye penetration. The findings showed that more recent premixed Bioceramic materials (BC-RRM and Bio-C Repair) had a much higher sealing capacity than MTA and Biodentine. Ability of sealing is essential to avoid bacterial ingress which is one of the leading causes of endodontic failures. It has been demonstrated that microleakage is directly related to treatment failure and the use of suitable root-end filling material is vital. MTA exhibited the highest level of microleakage in the current study and this is in line with other past researches that declared its shortcomings as lengthy setting time and difficulty in handling. MTA is a gold standard; however, it could have its sealing ability impaired by porosity and washout during initial setting. Biodentine was found to have a better sealing capacity than MTA. This can be explained by its reduced particle size, enhanced adaptation and quicker setting reaction, which improving marginal integrity. BC-RRM showed the best performance and Bio-C Repair came right behind, demonstrating better sealing properties of premixed bioceramics. These are hydrophilic materials, which create hydroxyapatite crystals, which create chemical bonding with dentin and enhanced sealing. Another micro-CT study has recently shown that bioceramic materials have a lower voids and porosity, which helps to improve sealing capability. Likewise numerous studies comparing MTA, Biodentine and bioceramic putty indicated that bioceramic putty demonstrated much less microleakage, which corroborates the results of the current study [11]. Compared with MTA, Rencher *et al.* found that BC-RRM putty and the paste+putty combination exhibited comparable sealing ability in an *ex vivo* Enterococcus faecalis leakage model, and SEM showed bacterial presence at the material-tooth interface for all groups, indicating no material was superior under the study conditions [12]. Moreover, many studies which have premixed bioceramic materials have better marginal adaptation and the sealing ability difference may not necessarily be statistically significant [13, 14]. These observations are consistent with the results of the current research, in which BC-RRM and Bio-C Repair demonstrated the lowest leakage, which validates the benefits of premixed formulations in minimizing variability in the operator. The bioactivity of bioceramics is another key consideration that results in the development of a mineral infiltration zone that enhances the seal with time [15, 16]. However, although there are benefits, other studies indicate that variations among bioceramic materials are not necessarily statistically significant as in the case of BC-RRM vs Bio-C Repair in this research. This implies that the two materials are clinically similar. The study has some limitations such as *in vitro* design, Use of dye penetration instead of bacterial leakage model and Absence of long-term assessment. In the future, the use of clinical trials and more sophisticated imaging modalities like micro-CT should be included in the research to provide more precise evaluation.

## Conclusion:

Bioceramic materials had a higher sealing capacity than MTA is. BC-RRM had minimum microleakage and then Bio-C Repair. There was no statistically significant difference in newer premixed bioceramics. Thus materials may be regarded as good substitutes of root-end filling.

## Figures and Tables

**Table 1:** Distribution of samples according to degree of microleakage

**Microleakage Score**	**MTA (n=18)**	**Biodentine (n=18)**	**BC-RRM (n=17)**	**Bio-C Repair (n=17)**
Score 0 (No leakage)	3 (16.7%)	5 (27.8%)	8 (47.1%)	7 (41.2%)
Score 1 ( ≤1 mm)	5 (27.8%)	7 (38.9%)	6 (35.3%)	6 (35.3%)
Score 2 (1-2 mm)	6 (33.3%)	4 (22.2%)	2 (11.8%)	3 (17.6%)
Score 3 ( >2 mm)	4 (22.2%)	2 (11.1%)	1 (5.8%)	1 (5.8%)

**Table 2:** Mean dye penetration (mm) in different groups

**Group**	**Mean ±SD (mm)**	**p-value**
MTA	1.82 ±0.42	
Biodentine	1.36 ±0.38	
BC-RRM	0.92 ±0.30	
Bio-C Repair	1.05 ±0.33	
ANOVA p-value		<0.001

**Table 3:** Intergroup comparison (post-hoc Tukey test)

**Comparison**	**Mean Difference**	**p-value**
MTA vs Biodentine	0.46	0.012
MTA vs BC-RRM	0.9	<0.001
MTA vs Bio-C Repair	0.77	<0.001
Biodentine vs BC-RRM	0.44	0.018
Biodentine vs Bio-C Repair	0.31	0.041
BC-RRM vs Bio-C Repair	0.13	0.210 (NS)

## References

[1] Junior JFSJ (2018). Braz Oral Res..

[2] Dioguardi M (2021). Heliyon..

[3] Srilakshmi J (2025). Bioscan..

[4] PrasadKumara PAAS (2025). Dent J (Basel)..

[5] Atmeh AR (2012). J Dent Res..

[6] Surana P (2024). Bioinformation..

[7] Kaur M (2017). J Clin Diagn Res..

[8] Pinto NMF (2015). Rev Odonto Cienc..

[9] Kwak SW (2023). J Endod..

[10] Hashem Q (2024). BMC Oral Health..

[11] AlbernazNeves J (2025). Sci Rep..

[12] Rencher B (2021). Restor Dent Endod..

[13] Vaiani L (2023). J Funct Biomater..

[14] Vijith V (2024). J Pharm Bioallied Sci..

[15] Saxena P (2013). Restor Dent Endod..

[16] Gangishetti S (2024). J Pharm Bioallied Sci..

